# Integration of fish culture and poultry rearing in transplanted rice for nutritional security in smallholder farms

**DOI:** 10.1038/s41598-020-67657-4

**Published:** 2020-06-29

**Authors:** Kathiresan Ramanathan, Vishnudevi Sangeeviraman, Prabakar Chandrahasan, Badri Narayan Chaudhary, Srikrishna Sulgodu Ramachandra

**Affiliations:** 1 Faculty of Agriculture, Annamalai University, Annamalainagar, Chidambaram, 608002 India; 2 Commercial Agriculture Alliance, Biratnagar, 56613 Nepal; 3 IKP Knowledge Park, Secunderabad, Telangana 500009 India

**Keywords:** Biotechnology, Plant sciences, Health care

## Abstract

Agriculture provides livelihood for 65% of Nepal’s population contributing for 27% of its GDP. Smallholder farms constitute 60% of farming segment. Distress farming, with inadequate minimum support price, subsidies and inadequate revenue generation force 29% of the general population to be malnourished. Farming system designs with integration of animal components would augment animal protein intake of these resource-poor populations, livelihood enhancement and sustainability of production system. On-farm field experiments in 75 participating farmers fields of Nepal with integration of fish culture and poultry rearing in transplanted rice showed appreciable (a significant) increase in nutritional status and livelihoods of participating farmers.

## Introduction

Agriculture provides livelihoods for 65% of Nepal’s Population accounting for 27% of its GDP^1^. Farming in Nepal Comprise 60% of small farm holdings with average holding size of less than 0.8 hectare^2^. The total cultivated area is 41,21,000 hectares^3^ and area under rice constitute 1.5 million hectares^4^. Distress farming, due to inadequate minimum support price and subsidies is predominant. These distress farming conditions and inadequate revenue generation force 29%^5^ of the general population to be malnourished. The farming constraints identified are poor rainfall distribution linked to monsoon based monocropping of rice, dismal economic returns from rice, inadequate or absence of diversification of farm components and exclusive dependence on agrochemical inputs. The prime remedial measure proposed to alleviate these constraints is integrated farming systems approach with integration of animal components. This would improve income generation from the farm through animal products, reduce agrochemical use with complimentary manuring and pest suppression from the animal components while enhancing diet diversity, which in turn would add to boosting of the nutritional status of these resource-poor farmers. A judicious combination of any one or more of the enterprises with cropping component was observed to result in better utilization of available resources through effective recycling of residues or wastes and offered employment to the family labour during off-season and made the farm a viable unit^6^. Integrated farming system with the components like pig, duck, fish and azolla was suggested to be more productive for rice dominated La Union region of Philippines^7^. The rice-fish system was observed to be a profitable technology and that adoption increased household income, labour absorption and better liquidity^8^. Main beneficial effects of rice-fish culture were related to environmental sustainability, system biodiversity, farm diversification and household nutrition^9^.

About 500–600 poultry birds provided adequate quantity of litter for manuring one hectare of waste area in the fish pond^10^. Integration of fish in rice fields increased dietary standards in terms of animal protein requirement of the poor rural households ^11^. The exotic broilers were replaced with indigenous chicks in vertically integrated homestead fish pond as a means of improving the income status of small scale farmers in Nigeria^12^. In the integrated fish-livestock farming, cheap fish feed and organic manures for fish pond recycled from livestock voidings reduced the cost of fish feed and chemical fertilizers. This indicated that integrated farming system was a Low-cost technology with high output^13^. Use of organic manures along with organic pest control in rice was demonstrated as a sustainable approach in rice farming with enhanced crop productivity, improved soil fertility status, increased economic return and reduced agrochemical input^14^. Integrating allied components with cropping system helped in effective water budgeting and better economic returns in lowland farming^15^.

Results of the studies at annamalai university, department of Agronomy with statistically laid out field experiments on Integrated Rice + Fish + Poultry Farming System comprising individual treatment plots of size 40 square meter, each with a fish trench of 1 m depth and 0.5 m width, running along the boundary of one side and a poultry cage installed at the center of each plot accommodating 4 broiler birds with wire mesh as the bottom and sides of the cages, showed that the system resulted in an increase in net income of Rs. 2,04,297 (US$ 2,701) over monocropping of rice and a cost–benefit ratio of 1:2.40^16^. This system was upscaled for adoption by farmers in four districts of the state of Tamilnadu in India and was named as Annamalai Rice + Fish + Poultry Farming System. Animal components integrated with the farming system viz., poultry and fish also enhanced the fertility level of lowland rice soil that was revealed by increased Post-harvest soil available Nitrogen, Phosphorus and Potassium^17^. This increase in soil fertility status was made possible by gradual addition of 17.7 t/ha of poultry manure, spread throughout the cropping period^18^. Integrating fish culture in rice also favoured management of weeds contributing for 20% of weed control^19,20^. Complementary weed control from rice + fish + poultry farming^21^ and reduced pest incidence^18,22^ have also been reported.

In Annamalai integrated farming system fish trenches running alongside any one of the border of rice fields of dimension 20 × 10 m accommodated fish polyculture comprising a stocking density of 5,000 fingerlings/ha of equal proportions of Catla, Roghu, Mrigal, Common carp and Grass carp. These fish trenches occupied 7.5% of the rice area (15 square meter), with a depth of 1 m, top width of 0.75 m and bottom width of 0.5 m. Broiler rearing is integrated in these rice fields @1 bird for every 10 square meter of rice area, using a poultry cage of floor size 1.8 × 1.2 m and 0.9 m height that accommodated 20 birds. These cages were randomly installed in the rice fields using concrete posts 2.4 m tall, 1.2 m interred inside the field, lifting the cages 1.2 m above. The broiler waste was reaching the rice field through the wire mesh bottom of the cages, and got dissolved, in the water retained in the field to a height of 5 cm, there by serving as a rice manure and feed for fish. Irrigation water that enters the field through one end and while reaching the other end with the flow served the purpose of spreading the poultry waste in the field. This design elided the laborious task of collecting the poultry waste for application to rice fields that stand apart in other conventional farming system designs, besides the purview of some wastage^17,18^.

## Materials and methods

**Plate 1:**
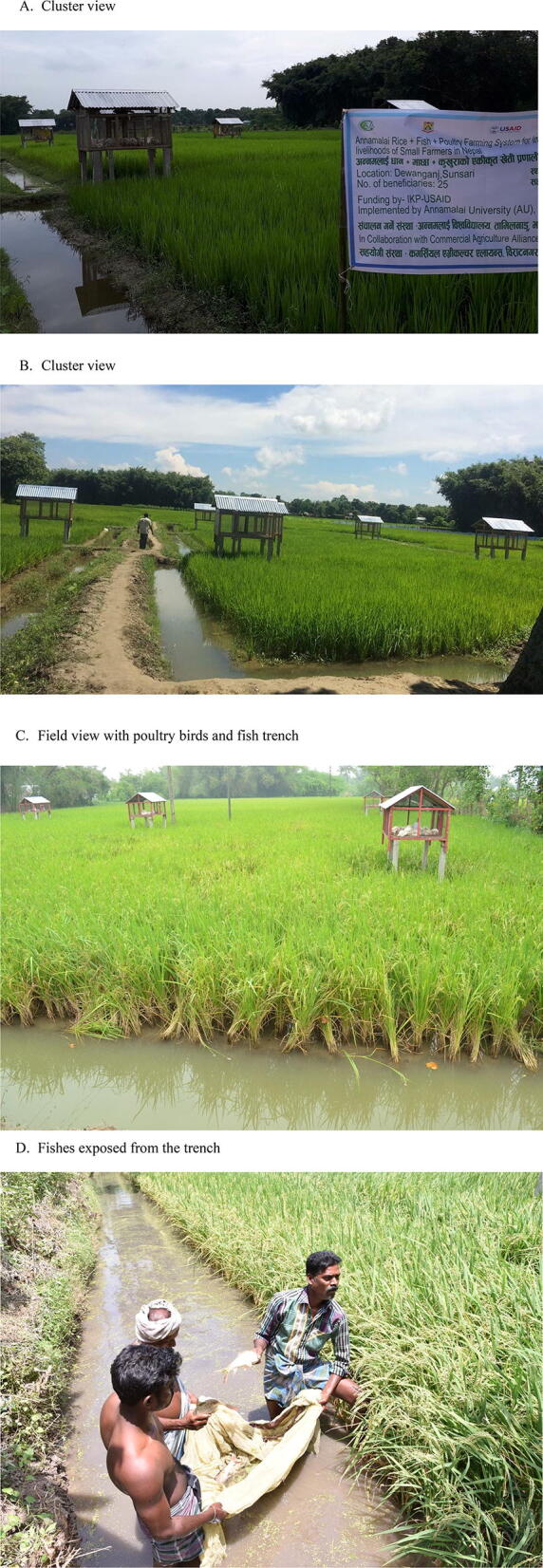
Annamalai Rice + Fish + Poultry farming system implemented in 75 selected small farm holdings of Nepal.

The Annamalai Rice + Fish + Poultry farming system was implemented (Table 1) in 75 selected small farm holdings of Nepal, from 2017 to 2019. (Plate 1: The participant farmers are informed and due consent obtained for publishing the images identified in the plates).

**Table 1:** Scheme of implementation.

Initiation	Activity	Completion
July, 2017	Identification of participating farmers	August, 2017
August, 2017	Installation of infrastructure for poultry and fish in rice fields	December, 2017
July, 2018	Rice nursery raising	August, 2018
August, 2018	Rice cropping along with poultry rearing and fish culture in rice fields	January, 2019

### Selection of target site and participating farmers

Sunsari district of Nepal was chosen as the target site and 75 farmers from the villages of Devanganj, Shankarpur, Kaptanguji and Sunsari were chosen as the participating development partners, as Sunsari district and the villages chosen exclusively cultivate rice under transplanted mode during the main cropping season. Further, these villages are predominated by resource-poor farmers with marginal farm holdings.

The participating 75 farmers were selected by adopting simple random sampling method.

### Technological intervention/novelty

The Annamalai Rice + Fish + Poultry system differs from the conventional integrated farming system.

This Annamalai model is different from conventional Rice + Fish + Poultry system demonstrated generally until now by other Agricultural Universities and Research Institutes. The conventional models have fish ponds excavated exclusively over 400 m^2^ area in one acre of rice field (4,000 m^2^). A poultry cage is installed in the fish pond and these two viz., poultry and fish components confine themselves to 400 m^2^, without directly supplementing the remaining 3,600 m^2^ of rice. Further, mostly layer birds are used in the poultry component. The bird’s waste that adds valuable organic manure has to be collected at the end of every farming season for application to rice. Inorganic agro inputs like pesticides and fertilizers are frequently used for rice, as rice has no direct integration with fish.

But in Annamalai Rice + Fish + Poultry model:

The poultry cages are installed in the rice fields straightaway, with the help of four concrete posts 2.4 m high, 1.2 m buried inside and 1.2 m protruding above, that lifts the cage above the crop canopy. The cage bottom is of wire mesh which leaves the poultry waste to reach the rice fields directly, wherein they get dissolved in standing water and serve both as crop manure as well as fish feed.

The fish trenches that accommodate the fishes, as a permanent shelter are 1 m deep and possesses a width of 1 m at the top and 0.75 m at the bottom and they run along the side of the rice field, occupying 7.5% of the rice fields. The fish fingerlings as a polyculture with Catla, Rogu, Mrigal, Common carp in equal proportions of a stocking density of 5,000 fingerlings ha^−1^ (considering the rice field dimension and not the trench dimension) are released after 15 days of transplanting rice seedlings. They swim into the rice fields and feed on the pests and weeds during morning and evening hours and take shelter in the trench during day time with sunny weather to avoid temperature fluctuation of shallow water column standing in the rice field.

The poultry cage dimension and poultry stocking density are optimized through rigorous experimentation. Cages are of dimension 1.8 m × 1.2 m × 0.9 m accommodating 20 broiler birds in each cage @ 1 bird/0.3 m^2^ of cage floor space and 10 m^2^ of rice field area. Larger cages and higher stocking density hamper crop growth because of shading and by increased volume of poultry litter per unit area that are acidic in nature, respectively.

Further, three to four generations of broiler birds within one rice cropping season, offers excellent revenue generation that enhances livelihood security of resource-poor farmers. In case of natural calamities such as flash floods wherein the crop could totally be damaged, this broiler meat output would offer solace and serve as a climate resilience mechanism.

Installations of infrastructure for adoption of Annamalai Rice + Fish + Poultry farming system in all the 75 farmers holdings, in area of 200 m^2^ of rice fields in the holdings of identified participating farmers were organized. Digging of fish trenches of 20 m^2^ in all the fields (20 m × 1 m), installation of cement concrete posts and poultry cages were done in phased manner.

The impact assessment questionnaire was prepared and the details were obtained from 75 farmers including men and women, before intervention as a baseline survey and again after intervention as final impact assessment survey.

The baseline survey and final impact assessment included annual household income in nepalese rupees (1 US$ = 121.29 NPR), house hold food intake per month with particular reference to poultry meat, fish meat, and vegetables along with farmer’s blood biochemical parameters such as haemoglobin count, serum albumin, folic acid and calcium, of the individual farmers adopting the technology. The blood biochemical parameters were analyzed in nationally accredited clinical laboratories, as instructed by the sponsors of the project.

The salient observations on household diet consumption particularly poultry meat, fish meat and vegetables intake per month, blood biochemical parameters such as haemoglobin, serum albumin, folic acid and calcium levels of farmers participating were subjected to paired t-test analysis using SPSS software. The number of pairs (N) was 75.

### Ethical approval

All these methods were carried out in accordance with relevant guidelines and regulations. The Experimental protocols were approved by Commercial Agriculture Alliance Human and experimental ethical committee of Nepal (No. CAA/HEC/NEPAL/03 dt. 03/10/2017). Data collection was taken up by Commercial Agriculture Alliance of Nepal after obtaining written informed consent from all subjects (Men and women, the participating farmers).

## Results and discussion

### Annual household income

Observation regarding annual household income is furnished in Table 2. The annual household income of the participating farming household were nepalese rupees 64,160 before the start of the intervention and the same rose to NPR 1,26,560, with the difference of NPR 62,400 that accounted to livelihood enhancement of 97%, almost double that of the baseline value. This increase is due to the revenue generated from the sale of broiler meat (180 kg of broiler meat produced from 200 m^2^ of holding), fish meat (15 kg from 200 m^2^ of holding) and increased rice grain yield (from the farm holdings of the participating farmers of 78 kg/2000 m^2^ of rice field), coupled with displaced agrochemical cost.

**Table 2:** Household annual income of participating farmer (in NPR).

S. no	Source	Income from source	Total income
**A. Before intervention (2018)**			
1	Rice monocropping	64,160	64,160
**B. After intervention (2019)**			
1	Rice cropping	65,200	1,26,560
2	Poultry rearing	56,110	
3	Fish culture	5,250	

### Poultry meat intake

**Figure 1:**
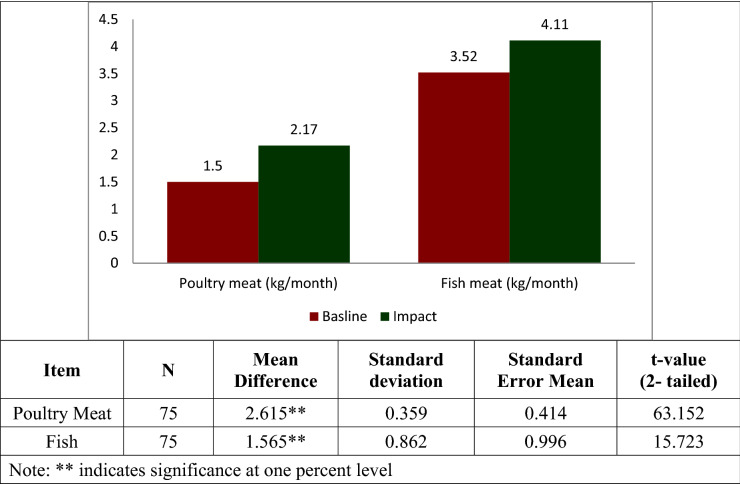
Consumption of poultry and fish meat (kg/month).

The pattern of household poultry meat intake per month observed is presented in Fig. 1. The intake pattern of farming households, participating through adoption of integrated farming system were 1.5 kg of poultry meat/month, during baseline survey i.e. before intervention. The poultry meat intake of participating households rose to 4.11 kg per month after the intervention (completion of broiler chicken rearing in the cages installed in their rice fields), with an increase by 2.74-folds. The increase in consumption of poultry meat by participating households are statistically significant as revealed by the paired t-test analysis. This is due to availability of 180 kg of poultry meat per household produced from their own fields, adopting integrated Rice + Fish + Poultry farming system. Besides, selling the major portion of meat that resulted in enhanced revenue generation, the farming households also were consuming some of this meat available from their own holding.

### Fish meat intake

The pattern of fish meat intake by the participating farming households is given in Fig. 1. The rate of fish meat intake per household per month was 2.17 kg before adoption of the technology and this increased to 3.52 kg with the farming households participating in technology adoption, with a 62% increase. The increase in consumption of fish meat by participating households are statistically significant as revealed by the paired t-test analysis.This is due to the production of fish meat from every participating farmers holding, besides the enhanced income generation from the innovative farming system adopted, that imparted better purchasing potential.

### Vegetable intake

**Figure 2:**
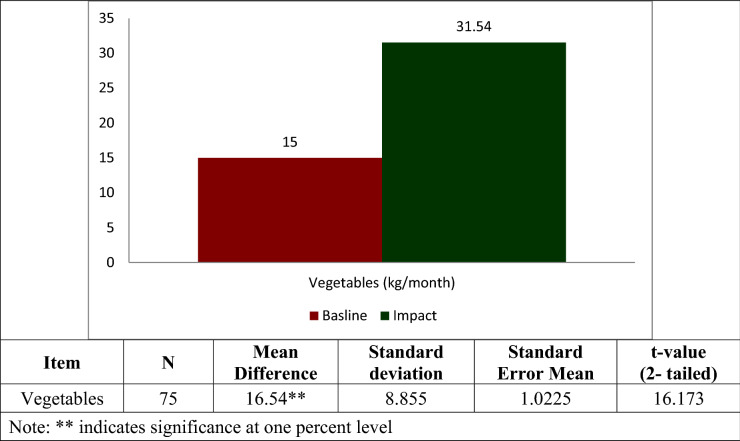
Consumption of vegetables (kg/month).

The trend of vegetable consumption as furnished in Fig. 2 also showed an increase in household consumption of vegetables per month, which is attributed to the enhanced household income that paves way for potential to buy more vegetables by the farmers adopting the technology. The increase in consumption of vegetables by participating households is statistically significant as revealed by the paired t-test analysis.

The results vividly reveal that there exists a significant difference in the consumption of poultry meat, fish meat and vegetables by those households adopting integrated farming system, between the after and before intervention scenarios. The mean values were significant at level, p = 0.01.

### Blood Biochemical Parameters

The observations regarding the blood biochemical parameters viz, haemoglobin, serum albumin, folic acid and calcium levels are presented in Table 3.

**Table 3:** Blood biochemical parameters of farmers adopting integrated Rice + Fish + Poultry farming system.

Haemoglobin (g/dl)		Folic acid (ng/ml)		Serum albumin (g/dl)		Calcium level (mg/dl)	
Baseline	Impact	Baseline	Impact	Baseline	Impact	Baseline	Impact
11.78	13.20	7.54	8.42	4.29	4.72	9.33	9.71

Item	N	Mean difference	Standard deviation	Std. error mean	t-value 2-tailed
**Results of paired t-test**					
Haemoglobin (g/dl)	75	1.41**	0.66	0.076	18.542
Folic acid (ng/ml)	75	0.87**	0.35	0.040	21.487
Serum Albumin (g/dl)	75	0.425**	0.25	0.0286	14.880
Calcium	75	0.375**	0.29	0.033	11.365

**Indicates significance at one% level.

It could be understood from the results that there exists a significant difference between the post and pre intervention scenarios interms of the level of blood biochemical parameters viz., haemoglobin, folic acid, serum albumin and calcium of the blood samples collected from farmers adopting integrated farming systems. The respective mean value of all the four clinical parameters were significant at level p = 0.01. The mean values of the levels of haemoglobin, folic acid, serum albumin and calcium in the Post-intervention scenarios were higher by 1.41 g/dl, 0.87 ng/ml, 0.425 g/dl and 0.375 mg/dl, respectively than the pre intervention scenario (Table 3). This significant difference is attributable to the increased poultry meat intake, fish meat intake and vegetables intake of the participating farmers in these villages, that was triggered by diversified farm production and enhanced income generation made possible by the adoption of integrated rice + fish + poultry farming system, replacing monocropping of rice.

## Conclusion

The study reveals the potential of Annamalai integrated Rice + Fish + Poultry farming system designed and implemented, in enhancing the nutritional status of resource-poor rice farmers of Nepal. This integrated Rice + Fish + Poultry farming system holds scope for upscaling in similar agroceologies of all Asian countries, predominated by smallholder rice farms towards ensuring nutritional security.
